# Hysteresis of heavy metals uptake induced in *Taraxacum officinale* by thiuram

**DOI:** 10.1038/s41598-021-99666-2

**Published:** 2021-10-11

**Authors:** Dorota Adamczyk-Szabela, Katarzyna Lisowska, Wojciech M. Wolf

**Affiliations:** Institute of General and Ecological Chemistry, Lodz University of Technology, Zeromskiego 116, 90-924 Lodz, Poland

**Keywords:** Ecology, Environmental sciences, Chemistry

## Abstract

Dandelion (*Taraxacum officinale*) yields active substances frequently used in herbal medicinal preparations. Its plantations are exposed to fungal plagues which pose a threat to herbal crops. The aim of this study was to evaluate the long time effects of a fungicide thiuram on dandelion growth and photosynthesis. Additionally, the manganese, iron, copper, zinc, cadmium, and lead uptake and transport were also investigated. Plants were cultivated under greenhouse conditions by the pot method in a universal flowering soil. The elements content in soil and plants were determined by the HR-CS FAAS spectrometer. Thiuram concentrations were established by the HPLC. Those analyses showed that almost 80% of thiuram decomposed within two weeks of its application. The photosynthesis indicators suggested, that plants were in good conditions and the fungicide supplementation facilitated plant growth. The latter could be prompted by thiuram acting as a sulfur rich chemical micro fertilizer. The hypothesis, that thiuram significantly affects heavy metals interactions in dandelion was proved by the one-way analysis of variance. Notable, metals uptake did not completely recover after fungicide decomposition for all investigated elements except iron We suggest to define this chemically induced, time-dependent heavy metals migrations in the soil–plant system as hysteresis of heavy metals uptake.

## Introduction

Despite remarkable progress in pharmaceutical chemistry herbal plants are still an important source of active substances used in many medicinal formulations. Notable, herbs quality is strongly associated with cultivation conditions. The latter together with the growing market of herbal products prompted exceptional development of herbal agriculture with special emphasis directed towards exceptional crops quality^1–3^. Herbal pharmaceuticals are usually administered over long time periods. Consequently, even very low doses of heavy metals and chemicals as present in plants can accumulate in the patient's body^4^.

*Taraxacum officinale* (dandelion) is a herbaceous perennial plant of the family *Asteraceae*. Remarkable, all parts of the plant have been used in the traditional medicine^5^. Dandelion contains several pharmacologically active compounds like, flavonoids, phenolic acids, terpenoids, triterpenes, and sesquiterpenes. According to the British Herbal Medicine Association^6^, its roots are useful hepatic stimulant, whereas leaves exhibit the diuretic and choleretic actions. Dandelion is a valuable indicator of heavy metals contamination and has been applied to evaluate As, Br, Cd, Co, Cu, Cr, Hg, Mn, Pb, Sb, Se and Zn bioavailability^7,8^. Kano et al.^9^ applied *Taraxacum officinale* to remove cadmium and zinc from soil by phytoremediation. Hammami et al.^10^ showed that, based on the rate of Cd(II) reduction in contaminated soil, dandelion was among the most effective weed species.

For years, the raw material of dandelion originated from natural, wild sources. However, the increasing demand prompted expansion of dedicated plantations which have been mostly established in France, Germany and Poland^11–14^. Like in efficient, product oriented agriculture fungal-induced plagues have posed a serious threat to high quality herbal crops and inflicted serious harvest losses^15^. Furthermore, spices and herbs used for production of pharmaceuticals should be free of pathogens and subjected to strict standardization procedures. Therefore, the effective protection of plantations with pesticides of well established, low toxicity is of primary value^16,17^. Namely, contact fungicides which can be applied before the herbs harvest, over time which ensures their complete degradation to safe metabolites are of particular importance.

Dithiocarbamates and especially thiuram (i.e. tetramethylthiuram disulfide), are widely used to control fungal plant diseases. Vegetables, fruits, herbs and decorative plants are primary targets^18^.

Thiuram activity originates from inhibition of the pyruvic dehydrogenase system at the fungal cells resulting in decrease of glucose and oxygen metabolism^19^. The degradation of thiuram in either soil or plant environments is a complicated process, which depends on diverse factors. Soil moisture, organic content and microbial activity are among the most important. Leading metabolites are dithiocarbamates, dimethylamine, and carbon disulfide^20^. The latter is particularly toxic to soil biota. Furthermore, there are evidences that thiuram has the ability to complex metal ions in the soil environment^21–23^ and affects heavy metals uptake by plants over the cultivation time. This problem is crucial for the consumers health and attracted concern of the European Commission which established maximum residue limits (MRLs) for common pesticides and heavy metals in either raw or processed food products^24^.

In this paper we examine dynamics of additive interactions which involve manganese, iron, copper, zinc and cadmium and are prompted by thiuram administered over green parts of dandelion. Experimental conditions were close to those which are applied during field cultivation. The decomposition of thiuram in soil and plant tissues was investigated by the HPLC. Additionally, heavy metals concentrations were determined simultaneously over time. The study follows our ongoing investigations on combined, additive effects in *Taraxacum officinale* plants cultivated in either normal or stress conditions^23,25,26^.

### Reagents

Thiuram standards of high purity were obtained from Sigma-Aldrich. The thiuram stock standard solution 200 µg/ml was prepared in acetonitrile and stored at 4 °C. Ultra-gradient HPLC grade acetonitrile, anhydrous magnesium sulfate, sodium chloride, sodium sulphate, trisodium citrate dihydrate and disodium hydrogencitrate sesquihydrate were purchased from Fluka (Steinheim, Germany). Primary secondary amine (PSA) were supplied by Supelco (Bellefonte, PA, USA).

## Methods

### Raw soil analysis

Soil was purchased at the garden store. It was a universal flowering soil from the company Hollas. Soil (25 samples) were dried in a well ventilated place and sieved through a 2-mm stainless steel sieve. Soil pH was established by the potentiometric method in 1 mol/dm^3^ potassium chloride solution^27^. The gravimetric method for the investigation of soil organic matter by the mass loss at 550 °C was used^28,29^. The bioavailable forms of metals were determined in 0.5 mol L^−1^ of hydrochloric acid extracts. 2.0000 g of soil (ground in a porcelain mortar and sifted through a 2 mm stainless steel sieve) were placed in plastic beakers and 100.0 ml of 0.5 L^−1^ HCl were added. Then the contents was stirred with a magnetic agitator for 0.5 h at a rate of about 40 rev/min and subsequently left until the next day. The solutions were then passed through a medium cellulose filter and the first part of the filtrate was rejected^30^.

The pseudo total metal content was determined in samples digested with the Anton Paar Multiwave 3000 closed system instrument. The mixture of concentrated HNO_3_ (6 mL) and HCl (2 mL) was applied (0.5000 g of soil). Metal concentrations were determined by the HR-CS FAAS with the contraAA 300 (Analytic Jena spectrometer).

### Soil sample preparation and soil analysis after thiuram supplementation

About 160 g of dried and sieved soil together with 6 mg of thiuram were placed in each of five plastic pots. The bioavailable metal forms were determined in 0.5 mol L^−1^ of hydrochloric acid extracts after 2, 4, 7 weeks since the fungicide application. Metal concentrations were measured by the HR-CS FAAS with the contraAA 300.

Thiuram concentration in soil were determined after 2, 4, and 7 weeks of its supplementation^31^. A 10 g portion of soil air dried, homogenized and sieved (2 mm) was weighted into a 250 ml conical flask, then 50 ml of acetonitrile was added. The content was vigorously shaken in a laboratory shaker for 15 min, centrifuged and transferred into the chromatograph (D-7000 HPLC Merck Hitachi) with UV detector. The ACE 5 C18 (15 cm × 4.6 mm) column was applied. The mixture of acetonitril and water (50%: 50%) was used at a flow rate 1 ml/min. The UV detector was set at 254 nm. Under above conditions the retention time for thiuram was 3.8 min. Calibration curve for thiuram was determined for the 0.5–4.0 µg ml^−1^ concentration range, *r*^2^ = 0.9991. Recovery studies were conducted by spiking thiuram at levels of 4.0; 10; 40; 200 µg g^−1^ with average recovery 99%, 102%, 101%, 101%, respectively.

### Plant material sample preparation and analysis

Dandelion plants were cultivated under laboratory conditions by the pot method^32^ from April to July 2019. The soil portions (160 g) were put into 20 plastic pots. Seeds of *Taraxacum officinale* L. were sown in an amount of 0.10 g (approximately 30 seeds) per pot. All pots were kept in a greenhouse at controlled temperatures 23 ± 2 and 16 ± 2 °C for day and night, respectively. The relative humidity was limited to 70–75%. The photosynthetic active radiation (PAR) during the 16-h photoperiod was restricted to 400 μmol m^−2^ s^−1^. All plants were watered with the same amount of deionized water every 3 days. Thiuram was applied (6 mg thiuram per pot) to plants, one month after they had been sown. Herbs were harvested in periods of 2, 4 and 7 weeks after fungicide treatment. The above-ground parts of plants were cut, while the roots were separated from soil by washing and rinsing with distilled water. The entire harvest was oven-dried at 45 °C (SML 30/250 Zelmed, Poland) to a constant weight, homogenized, and grounded.

#### Statement on guidelines

Experimental research and feld studies on cultivated plants comply with relevant institutional, national, and international guidelines and legislation.

### Determination of heavy metals in *Taraxacum officinale*

The dried roots and above-ground parts of dandelion (0.5 g sample) were digested in a microwave mineralizer (Anton Paar Multiwave 3000 closed system instrument) with concentrated HNO_3_ (6 mL) and HCl (1 mL) acid solutions. Metal contents were measured by the HR-CS FAAS with the contraAA 300 and GAAS with the Scientific Equipment GBC 932 plus and GBC, SensAA spectrometers, respectively.

### Quality control and quality assurance

The reliability of the analytical procedures was evaluated with the certified reference material INCT-MPH-2, i.e. a mixture of selected Polish herbs^33^ (Table S1). Limits of detection (LOD) and quantification (LOQ) determined for HR-CS FAAS are given in Table S2. LOD was computed from 10 successive measurements of the signal corresponding to the blank, which was prepared in the same way as the plants samples. Limits of quantification were the lowest concentrations determined with relative standard deviations not exceeding 10%, of triplicated LOD.

### Dandelion plant growth and its physiological activity

Plant heights were measured from the soil surface up to the highest part of the leaf. Index of chlorophyll was determined by measuring the leaf absorbance in the red and near-infrared regions with the Konica Minolta SPAD-502Plus, Tokyo, Japan. Activity of net photosynthesis, stomatal conductance, intercellular concentration of carbon dioxide, and transpiration were determined with the gas analyzer apparatus CIRAS-3 (Portable Photosynthesis System, Amesbury, MA, USA)^34,35^. All measurements were replicated five times on separate dandelion plants.

### Determination of thiuram residues in plant

The QuECheRS extraction method was applied to plant samples^36^. Approximately 9 g of the homogenized plant tissues were placed into a 50 mL polypropylene tube. Then, 10 mL of acetonitrile was added and the mixture was hand-shaken for 5 min. Four grams of anhydrous magnesium sulfate, 1 g of sodium chloride, 1 g of sodium citrate tribasic dehydrate and 0.5 g sodium citrate dibasic sesquihydrate were added, and the mixture was immediately shaken for 5 min, and further centrifuged for 5 min at 4300 rpm. Then, the acetonitrile phase was transferred to a polypropylene tube containing MgSO_4_ and PSA, was shaken for 2 min and was centrifuged for 5 min at 4000 rpm. The final extract was subjected to the HPLC analysis as above.

### Data analysis

All analyses were repeated five times. Bartlett's and Hartley's tests were applied to check the equality of variance (STATISTICA 10 PL package). Normality of the data sets was evaluated using the Shapiro–Wilk test^37,38^. One-way ANOVA was followed by the post-hoc Tukey’s test^39^.

## Results

### Analysis of soil

**Figure 1:**
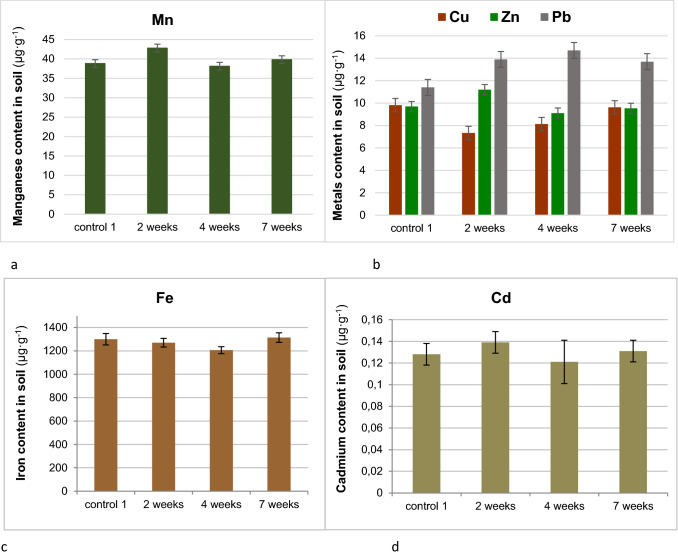
Manganese (**a**), copper, zinc, lead (**b**), iron (**c**) and cadmium (**d**) bioavailable forms in soil supplemented by thiuram displayed against the thiuram contact time (weeks). For clarity, the manganese, iron and cadmium contents are displayed on separate pictures.

Manganese, iron, copper, zinc, cadmium and lead contents in raw, untreated soil are in Table 1. Additionally, their bioavailable forms were being determined periodically after 2, 4 and 7 weeks since the thiuram application to soil (Fig. 1).

**Table 1:** Metals content in soil (mean ± SE, *n* = *5)*.

Metal content (µg g^−1^)	Mn	Fe	Cu	Zn	Cd	Pb
Pseudo total	41.2 ± 1.1	1762 ± 51	15.4 ± 0.3	12.5 ± 0.7	0.940 ± 0.130	14.6 ± 0.8
Bioavailable	38.8 ± 0.5	1300 ± 49	9.8 ± 0.17	9.69 ± 0.52	0.128 ± 0.010	11.4 ± 0.7

### Influence of thiuram on Mn, Fe, Cu, Zn, Cd and Pb contents in dandelion

**Figure 2:**
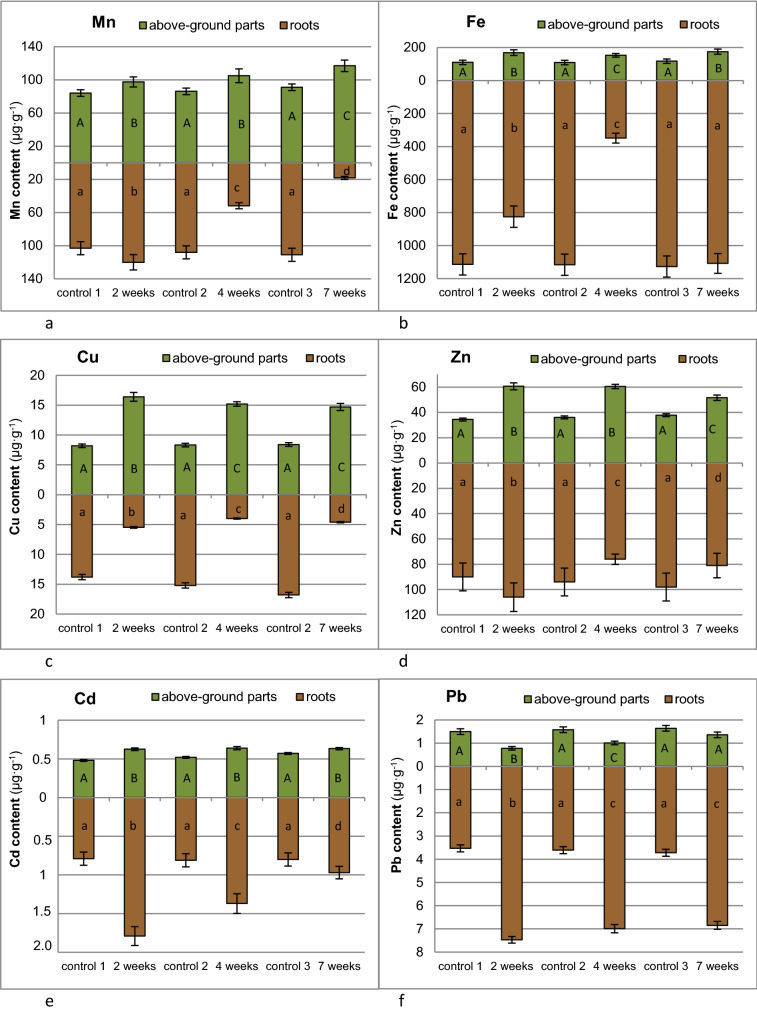
Manganese (**a**); iron (**b**); copper (**c**); zinc (**d**); cadmium (**e**) and lead (**f**) contents in roots and aboveground parts of dandelion plant displayed against the thiuram contact time. Control data as indicated with numbers 1, 2, 3 refer to the same time intervals as samples subjected to thiuram treatments, namely 2, 4 and 7 weeks, respectively. Specific letters illustrate the statistically significant differences as computed with the Tukey’s HSD test (*p* = 0.95), roots and above-ground parts are treated independently.

Metal contents in either roots or above-ground parts of dandelion cultivated in soil supplemented with thiuram are summarized in Fig. 2. Plants grown in thiuram free environment mostly accumulated heavy metals in roots. Thiuram supplementation changed this picture substantially. The highest concentration of metal in roots was observed for Fe and Pb and to a lesser extent for Zn and Cd. The reverse effect has been detected for Cu and Mn which were mostly accumulated in green parts of the dandelion plant.

Initial fungicide doses were as recommended by the manufacturer. Thiuram concentrations in either soil or dandelion plants determined over time (Table 2) showed that 80% of fungicide decomposed within two weeks of its application. The influence of thiuram contact time on heavy metal uptake by dandelion plants was assessed by the one-way ANOVA at the 0.95 probability level (Table 3) and followed by the post-hoc Tukey’s test^39^. The null hypothesis was, whether thiuram supplementation affects migration of metals from soil to roots and subsequently to above-ground parts of the plant for a particular time of cultivation. Namely, 2, 4 and 7 weeks after the fungicide supplementation. Those calculations demonstrated that thiuram could contribute to metals uptake in dandelion.

**Table 2:** Thiuram concentration in soil and dandelion plant over contact time (mean ± SE, n = 5).

Thiuram contact time weeks	Thiuram concentration	
	Soil (µg g^−1^)	Plant (µg g^−1^)
control	37.5 ± 1.9	600 ± 27
2	7.52 ± 0.41	72.0 ± 3.8
4	3.45 ± 0.20	42.0 ± 2.4
7	*	*

*Below the detection limit.

**Table 3:** One-way ANOVA for manganese, iron, copper, zinc, cadmium and lead contents in dandelion plant across the thiuram contact time (weeks).

	Mn	Fe	Cu	Zn	Cd	Pb
Roots	F = 213.3205 *p* = 4.14 × 10^–13^	F = 163.2660 *p* = 3.30 × 10^–12^	F = 1413.6520 *p* = 1.33 × 10^–19^	F = 22.2874 *p* = 5.87 × 10^–6^	F = 70.2164 *p* = 1.99 × 10^–9^	F = 506.2015 *p* = 4,64 × 10^–16^
Above-ground parts	F = 17.4783 *p* = 2.65 × 10^–5^	F = 15.9513 *p* = 4.57 × 10^–5^	F = 194.1126 *p* = 8.62 × 10^–13^	F = 148.6561 *p* = 6.80 × 10^–12^	F = 80.3759 *p* = 7.29 × 10^–10^	F = 41.5007 *p* = 8.95 × 10^–8^

Critical Snedecor’s F value is F_cryt_ = 3.2389.

### Thiuram effects on the photosynthetic apparatus

The plant health status was examined by five photosynthesis indicators i.e. index of chlorophyll content in leaves, the activity of net photosynthesis (P_N_), stomatal conductance (G_s_), transpiration rate (E) and intercellular concentration of CO_2_ (C_i_) (Table 4). Heights of dandelion plants were measured for investigated series. Those parameters unequivocally suggested that dandelion plants were in proper growing conditions. Increase of the plant growth was observed after thiuram supplementation (14–50%). Notable, heights of plants were consistent with photosynthesis indicators. As expected, the only exception was intercellular CO_2_ concentration which was decreasing upon the photosynthesis intensification. It means that photosynthesis acceleration is larger than that of stomatal conductance. Chlorophyll content in samples treated with thiuram was 10% higher than in controls over the whole cultivation time.

**Table 4:** The height of plant and photosynthesis indicators (mean ± SE, *n* = *5*).

Parameters	Thiuram contact time					
	2 week		4 week		7 week	
	Control 1	Sample 1	Control 2	Sample 2	Control 3	Sample 3
Height of plant (cm)	5.20 ± 0.30	7.40 ± 0.40	6.30 ± 0.40	9.50 ± 0.20	9.80 ± 0.30	11.2 ± 0.50
Index of chlorophyll	18.3 ± 0.9	20.2 ± 1.1	19.5 ± 1.4	21.7 ± 0.8	22.4 ± 1.8	24.8 ± 2.4
P_N_ (µmol CO_2_ m^−2^ s^−1^)	5.33 ± 0.42	7.11 ± 0.50	5.85 ± 0.50	7.23 ± 0.61	6.35 ± 0.35	8.15 ± 0.65
G_s_ (mmol H_2_O m^−2^ s^−1^)	342 ± 8	398 ± 6	349 ± 9	402 ± 7	351 ± 8	409 ± 7
E(mmol H_2_O m^−2^ s^−1^)	1.58 ± 0.20	1.93 ± 0.23	1.86 ± 0.18	2.08 ± 0.21	1.97 ± 0.19	2.11 ± 0.20
C_i_ (µmol CO_2_ mol^−1^air)	328 ± 7	308 ± 6	341 ± 8	311 ± 10	339 ± 9	309 ± 7

### Metals uptake by dandelion plant under thiuram application

Metals uptake from soil to plant was evaluated by their transfer coefficients (TC) which are defined as ratio of particular element concentration in plant roots to its content in the soil^40,41^. Metal distribution inside the plant body was assessed by translocation factors (TF) which are ratios of element concentration in above-ground part of the plant to that in roots^42–44^. Moreover, bioaccumulation factors (BAF) express transport of metals from soil to aboveground-parts. Relevant TCs, TFs and BAFs are summarized in Table 5. Series of those coefficients given in ordered way provide useful information on metals migrations and associated interactions which may affect plant tissues^45^.

**Table 5:** Series of metals ordered according to decreasing transfer coefficients (TC’s), translocation factors (TF’s) and bioaccumulation factors (BAF’s) calculated for *Taraxacum officinale* cultivated in soil with thiuram supplementation. Numerical data are in parenthesis.

Treatments (weeks)	Transfer coefficients
0*	**Zn** (7.20) > **Mn** (2.05) > **Cu** (0.90) > **Cd** (0.84) > **Fe** (0.63) > **Pb** (0.24)
2	**Zn** (8.48) > **Mn** (2.91) > **Cd** (1.90) > **Pb** (0.51) > **Fe** (0.47) > **Cu** (0.36)
4	**Zn** (6.08) > **Cd** (1.46) > **Mn** (1.26) > **Pb** (0.48) > **Cu** (0.26) > **Fe** (0.20)
7	**Zn** (6.50) > **Cd** (1.03) > **Fe** (0.63) > **Pb** (0.47) > **Mn** (0.44) > **Cu** (0.30)
**Translocation factors**	
0*	**Mn** (0.82) > **Cd** (0.61) > **Cu** (0.59) > **Pb** (0.42) > **Zn** (0.38) > **Fe** (0.10)
2	**Cu** (2.99) > **Mn** (0.81) > **Zn** (0.57) > **Cd** (0.35) > **Fe** (0.20) > **Pb** (0.10)
4	**Cu** (3.81) > **Mn** (2.02) > **Zn** (0.80) > **Cd** (0.47) > **Fe** (0.44) > **Pb** (0.14)
7	**Mn** (6.43) > **Cu** (3.19) > **Cd** (0.65) > **Zn** (0.64) > **Pb** (0.20) > **Fe** (0.16)
**Bioaccumulation factors**	
0*	**Zn** (2.75) > **Mn** (2.04) > **Cu** (0.53) > **Cd** (0.51) > **Pb** (0.10) > **Fe** (0.06)
2	**Zn** (4.85) > **Mn** (2.37) > **Cu** (1.06) > **Cd** (0.66) > **Fe** (0.10) > **Pb** (0.05)
4	**Zn** (4.84) > **Mn** (2.55) > **Cu** (0.99) > **Cd** (0.68) > **Fe** (0.09) > **Pb** (0.07)
7	**Zn** (4.13) > **Mn** (2.83) > **Cu** (0.95) > **Cd** (0.67) > **Fe** (0.10) > **Pb** (0.09)

*Control data are indicated with the null contact time, they refer to untreated plants and were collected just before the thiuram treatment.

## Discussion

Analysis of soil used in this study clearly indicated its acidic (pH = 5.2) and organic character (organic matter content was 90%)^27–29^. Manganese, iron, copper, zinc, cadmium and lead contents in raw, untreated soil (Table 1) showed that, according to either international^46^ or Polish^47^ standards soil was not contaminated by these metals.

The highest impact of thiuram on bioavailable forms of investigated metals in the soil was visible within two weeks after the fungicide application. In particular, respective concentrations of Mn, Zn, Pb and Cd were increased by 10–20% as compared to the control while 25% decrease was observed for Cu. Iron content was kept almost unchanged. After 7 weeks bioavailable metal contents returned to their original values. The only exception was Pb which enjoyed substantial mobility in soil over the seven weeks period.

Soil fungi and microorganism are to a large extent responsible for the heavy metals immobilisation in soil^48^. Thiuram destroys fungal cells and facilitates release of bioavailable heavy metals to the soil environment. This process was visible for Mn, Zn, Pb and Cd. Iron content in soil is one or two orders of magnitude larger than those of the latter metals. Therefore, effects induced by fungi affect only a relatively small fraction of bioavailable Fe. Copper is prone to complexation by thiuram metabolites like dithiocarbamates and dimethylamine what reduces its bioavailable forms substantially.

The uptake of pesticides and heavy metals by plants may be either enhanced or hampered by their associated interactions. In this study we unequivocally demonstrated, that thiuram has affected metals content in *Taraxacum officinale*. In particular, it increased Zn, Mn, Cu, Cd and Fe accumulation in above-ground parts of dandelion, while the opposite effect was observed for Pb. It is a toxic metal and its migration to the upper part of the plant is restricted^7^. Within the plant it is usually transported in a passive way. Additionally, in the presence of hydrogen sulfide it may be transformed to galena (PbS) which is hardly soluble. On the other hand, thiuram decomposition yields dimethylamine, which can act in a dual, contradictory role as either metal complexing agent or a base which reduces the rhizosphere acidity and subsequently the metal bioavailability.

The highest metal accumulation from soil by either plant roots (TC in the range 6.08–8.48) or its above-ground parts (BAF in the range 2.75–4.85) was observed for Zn at all time intervals after the fungicide addition. In general, above-ground parts were the least prone to metal migrations variations. Especially, BAFs of Zn, Mn, Cu and Cd were in the same orders during time of the study while Pb and Fe shared last or next to last positions in the series.

Thiuram supplementation changed order of TF series for all investigated metals. In particular, this treatment significantly accelerated copper and iron transport from roots to above-ground parts. On the contrary, their uptake from soil to root was hampered as indicated by significant decrease of TC determined two weeks after thiuram supplementation. Opposite to iron, the TC of copper did not recover to original value after thiuram decomposition. Bearing in mind that either Cu or Fe are essential elements for the plant growth^49^, their reduced availability in roots combined with the increasing demand of the growing above-ground parts prompted TF increase. A similar situation was observed for manganese, which uptake by roots from soil was significantly reduced within either 4 or 7 weeks after thiuram supplementation. Its migration from roots to above-ground parts was facilitated by a fungicide and led to increased accumulation of Mn in above-ground parts of the plants. Root uptake of both toxic metals i.e. Cd and Pb was accelerated by thiuram. The highest TC were observed after 2 weeks of fungicide supplementation and were not substantially reduced after either 4 or 7 weeks of cultivation. However, their further migration to above-ground parts was hampered as indicated by the low relevant TF values. Presumable, the latter effect results from well recognized stress mitigation strategies as adopted by plants^50^.

Thiuram increased Zn, Mn, Cu, Cd and Fe accumulation in above-ground parts as indicated by BAF. Reverse effect has been visible for Pb, which content in above-ground parts was reduced from 1.50 to 0.78 µg g^−1^ within 2 weeks after the thiuram treatment. Recovery to original values was observed after subsequent 4 weeks. Cadmium, manganese and iron concentrations have been less prone to changes generated by thiuram. They promptly stabilized after moderate increase within the first two weeks after fungicides applications.

Fungicides are group of chemical substances with diverse structure and properties. Mechanisms of their mobility in soil are far from being thoroughly understood^51^. Notable, the soil properties and environmental conditions are among the major factors which affect their persistence and biological activity. Apparently, the impact of heavy metals cannot be neglected. Thiuram solubility in water is very limited indeed (30 mg L^−1^, 20 °C) and according to Thomas^52^ it decomposes in soil by hydrolysis aided by photodegradation and microbial action. This process is prompted by acidic conditions and proceeds significantly slower in the basic soil environment^53^. Additionally, organic matter and clay in the soil are likely to adsorb thiuram and further hamper its uptake^31^. Especially, humic acids exhibit the well-recognized ability to form stable complexes with heavy metals, which affect metals mobility and rise their retention in organic soils^54^. On the other hand, Gupta et al.^55^ suggested that high concentration of humic acids has increased the rate of thiuram decay and has limited its persistence.

Our experiments indicate that major thiuram decomposition proceeds within two weeks while its traces are visible in soil over four weeks. Seven weeks period was sufficient for the complete fungicide decay. These data are quite consistent with the work of Sherif et al.^31^ who investigated thiuram degradation in heavy clay soils at pH = 7.8 and determined that 90% of fungicide decomposed within four weeks. Unfortunately, the number of publications on the thiuram degradation in soil and plant environments is quite limited indeed.

In this study heavy metals uptake by dandelion did not recover after fungicide decomposition for all investigated elements except iron. Similar hysteresis of plant responses to heavy metals was already detected by us^23,56,57^ but we were reluctant to name it directly. In plant systems neither synergistic or antagonistic interactions of heavy metals and pesticides over time have been reported so far^58,59^. Therefore, the term hysteresis defined as the dependence of particular system on its history may be useful for future research. This effect is particularly well visibly for manganese and copper. Migration of these essential metals from roots to above-ground parts remained high (TF_Mn_ = 6.43, TF_Cu_ = 3.19) over seven weeks after the fungicide treatment. It is well recognized, that manganese in plant environment may exist in diverse forms, which are strongly pH dependent while predominant copper species are CuOH^+^ and Cu_2_(OH_2_)_2_^2+^ complexes with organic chelates^7^. Furthermore, only divalent cations are prone to the root uptake and subsequent transport to the above-ground parts. We speculate, that dimethylamine, which is an important metabolite of thiuram and forms stable complexes with either Mn^2+^ or Cu^2+^ cations^60,61^ may restrain their availability to the plant. Manganese is involved in chlorophyll synthesis and water splitting which initiates the conversion of carbon dioxide to carbohydrates^62^. Copper is cofactor of plastocyanin which is an important electron carrier^63^. Both metals are crucial for the development of photosynthesis. To overcome the resulting deficit of available manganese and copper dandelion plants accelerate their transport from roots to above-ground parts as indicated by the high translocation factors and relatively low Mn and Cu concentrations in roots.

Fungicide supplementation enhanced photosynthesis and facilitated dandelion plant growth as shown in Table 4. On the contrary, Yüzbasioğlu and Dalyan^64^ observed that photosynthesis in leaves of tomato (*Solanum lycopersicum* Mill.) cultivated in hydroponic Hoagland solution was reduced after thiuram. Thiuram is a sulfur rich chemical entity. Its decomposition yields carbon disulfide which is further oxidized to sulfates^65^. The latter are important source of plant available sulfur^66^ which deficiency reduces nitrogen usage and plant growth^67^. Therefore, thiuram facilitates photosynthesis at concentrations used in agriculture and in this work. On the other hand, high fungicide levels may affect plant metabolism as it was reported by Yüzbasioğlu and Dalyan^64^.

## Conclusion

In summary, thiuram affects dandelion plant development and heavy metals uptake in a number of ways which significantly depend on the fungicide doses. This feature makes it an promising agent for the selective metal enrichment. However, further studies are clearly needed to determine how thiuram may be successfully facilitate enhanced soil remediation strategies. Particular attention should be paid to the relationship between thiuram, copper and manganese. The latter metals play a significant role in the photosynthesis process. Low doses of fungicide supports the photosynthesis and plant growth. Nowadays, highly efficient technologies for fuel desulfurization reduced anthropogenic sulfur in the environment and prompted the soil sulfur deficit. In these circumstances, thiuram may act in a dual role as a fungicide or a sulfur rich micro fertilizer. Obviously, we should bear in mind that only controlled and smart usage of pesticides act against decline of our planet biodiversity.

## Supplementary Information

Supplementary Information.
