# Supplementary material for: Hysteresis of heavy metals uptake induced in Taraxacum officinale by thiuram
Source: Sci Rep. 2021 Oct 11;11:20151. doi: 10.1038/s41598-021-99666-2 (PMC8505632; doi:10.1038/s41598-021-99666-2)
Supplement: Supplementary file 1 — Supplementary Information. [file 41598_2021_99666_MOESM1_ESM.pdf]

Hysteresis of Heavy Metals Uptake Induced in Taraxacum officinale by Thiuram

Scientific Reports

Dorota Adamczyk-Szabela[1,]*, Katarzyna Lisowska[1] and Wojciech M. Wolf[1,]

[1]Lodz University of Technology, Institute of General and Ecological Chemistry,
90-924 Lodz, Zeromskiego 116, Poland

***Correspondence: dorota.adamczyk@p.lodz.pl
Katarzyna Lisowska, e-mail: katarzyna.lisowska@edu.p.lodz.pl
Wojciech M. Wolf, e-mail: wojciech.wolf@p.lodz.pl

Table S1. Metals concentration in the certified reference material (*p* = 0.95; n = 5)

**Metal**	**Certified value μg g^{-1}**	**Found μg g^{-1}**	**Recovery %**
**Manganese**	191 ± 12	196 ± 7	103
**Copper**	7.77 ± 0.53	7.53 ± 0.38	97
**Zinc**	33.5 ± 2.1	34.2 ± 0.7	102
**Cadmium**	0.199 ± 0.015	0.206 ± 0.007	103
**Lead**	2.16 ± 0.23	2.13 ± 0.13	98

Table S2. Limits of detection and limits of quantification, relevant units are given in parentheses.

**Metals**	**λ (nm)**	**LOD**	**LOQ**
Mn ($mg \cdot L^{-1}$)	279.482	0.009	0.027
Fe ($mg \cdot L^{-1}$)	248.327	0.056	0.168
Cu ($mg \cdot L^{-1}$)	324.754	0.010	0.029
Zn ($mg \cdot L^{-1}$)	213.857	0.057	0.172
Cd ($mg \cdot L^{-1}$)	228.802	0.006	0.017
Pb ($\mu g \cdot L^{-1}$)	217.000	2.786	8.358
